# Stem Cell Therapy and Its Significance in Pain Management

**DOI:** 10.7759/cureus.17258

**Published:** 2021-08-17

**Authors:** Jaskamal Padda, Khizer Khalid, Ujala Zubair, Hussam Al Hennawi, Jayant Yadav, Abdulelah H Almanie, Krutagni Adwait Mehta, Fahriba Tasnim, Ayden Charlene Cooper, Gutteridge Jean-Charles

**Affiliations:** 1 Internal Medicine, JC Medical Center, Orlando, USA; 2 Internal Medicine, Avalon University School of Medicine, Willemstad, CUW; 3 Internal Medicine, Advent Health & Orlando Health Hospital/JC Medical Center, Orlando, USA

**Keywords:** stem cell therapy, chronic pain, osteoarthritis, neuropathic pain, discogenic back pain, mesenchymal stem cells

## Abstract

Pain management has always been a challenging issue, which is why it has been a major focus of many rigorous studies. Chronic pain which typically lasts for more than three months is prevalent at an astounding rate of 11% to 19% of the adult population. Pain management techniques have gone through major advances in the last decade with no major improvement in the quality of life in affected populations. Recently there has been growing interest in the utilization of stem cells for pain management. Advancement of stem cell therapy has been noted for the past few years and is now being used in human clinical trials. Stem cell therapy has shown promising results in the management of neuropathic, discogenic back, osteoarthritis, and musculoskeletal pain. In this article, we will discuss the role of stem cells in the pain management of the aforementioned conditions, along with the mechanism, adverse effects, and risks of stem cell therapy.

## Introduction and background

The term “chronic pain” refers to pain quality that remains persistent after the healing process or is present without the existence of tissue damage, which typically lasts more than three months [1]. This type of pain is a consequence of psychologic, biologic, and social factors in which the evaluation and management usually require a multifactorial approach [2]. In developed countries, about 30% of patients complain of moderately severe to severe pain which has lasted longer than six months [3]. A noted increment in chronic pain prevalence is correlated to adult life, estimating it to be at a rate of 11% to 19% [1]. On the other hand, the term “stem cells” points to the cells that are undifferentiated and have the capability to differentiate and replicate into variable types of tissue. Back in 1998, the initial cultivation of embryonic stem cells derived from a human was successfully established. Thereafter, stem cell attraction and interest have been growing. The transplantation of stem cells was applied just recently in the therapeutic measures of pain, as it has shown promising outcomes for the management of multiple disease entities including neuropathic pain, degenerative diseases of the joints, musculoskeletal pain that is unresponsive to the standard medical treatment, and intervertebral disc (IVD) disease [4].

## Review

Stem cell therapy

Stem cells are defined as undifferentiated cells capable of perpetual self-renewal and have the ability to differentiate into specialized cell types. During the embryonic stage of development, these cells play an important role in forming organs, and in adults, they help in organ repair as well as renewal of tissue functions. A stem cell is comprised of many distinct cell types, namely embryonic, adult, and induced pluripotent stem cells (iPSCs). Embryonic stem cells can be obtained from the inner cell mass of blastocysts and are capable of differentiating into all three germ layers. Adult stem cells can be categorized according to the tissue of origin, such as placenta and umbilical cord stem cells, hematopoietic stem cells, bone marrow-derived mesenchymal stem cells (MSCs), and adipose-derived MSCs. They are present in all tissues of the body and are important for repair and renewal when tissues or organs are damaged [5]. Adult stem cells were recently induced to develop into a pluripotent state in cells called iPSCs. These cells share some of the same characteristics as embryonic stem cells such as proliferation, morphology and gene expression. This was made possible by retroviruses, which were used to carry genes for transcription factors in adult cells [6]. 

Furthermore, stem cells can be classified based on their ability to differentiate. Totipotent cells are found when the fertilized ovum starts to divide and can differentiate into any form of cells to build organs. Pluripotent cells are generally in the form of embryonic stem cells or iPSCs and are capable of differentiating into any cell type but cannot form complete organs. However, differentiation into specific cell types remains very poor. It is still a subject of immense interest with regards to what constitutes the optimal substrate and environment for differentiation into specific cell types. Studies in animal models using PSCs or embryonic stem cells resulted in the formation of unusual solid tumors called teratomas resulting in early setbacks of human trials. However, over the years PSCs have been modified to limit this unusual proliferative capacity and have been successfully used to treat animals with conditions like diabetes, acute spinal injury, and visual impairment [5].

Multipotent stem cells are found in the form of adult cells and have the plasticity to become all the progenitor cells for a particular germ cell layer or they could be restricted to differentiate into one or two specialized cell types depending on the substrate provided. One such example is how retinoic acid has been used to induce MSCs into neuronal cells [7]. Additionally, the lack of immunogenicity and no ethical jurisdiction makes them best suited for use in humans [8]. These qualities make MSCs an ideal candidate for clinical application.

Different methods depending on the site can be used to harvest stem cells. Bone marrow stem cells can be harvested directly from blood, bone marrow (iliac crests/sternum) or the umbilical cord. Adipose tissue stem cells can be harvested by liposuction or excision of adipose tissue. Adipose tissue samples are digested with the help of collagenase and then they are centrifuged. This separates the precipitate layer containing adipose stem cells at the bottom and is called the stromal vascular fraction [7].

How does it work?

MSCs can rapidly divide and repair damaged cartilage making them attractive for repairing damaged tissues. MSCs have several advantages. They can be harvested from one’s own body and are easy to prepare. Further, they are immunoprivileged, meaning they do not elicit a significant immune response. First off, tissue engineering is used to prepare scaffolds to generate functional tissues for the replacement of damaged tissues. Harvested MSCs are then seeded into the scaffolds where they begin their function. Direct engraftment of the host is one of the main working mechanisms of MSCs. MSCs are initially guided by the signaling cascades initiated at the sites of injury. As the platelets aggregate, they release cytokines which activate an influx of macrophages and neutrophils to the site of injury. The permeability of blood vessels at the injury site increases, setting the stage for homing of MSCs, and therefore increasing the aggregation of MSCs [4].

Besides direct engraftment, MSCs release a number of trophic factors in a paracrine fashion including growth factors like TGF-beta, factors inducing angiogenesis, transcription factors, and cytokines like PGE2, IL-2 and IL-6 [4]. In fact, studies have shown that paracrine function may be even more significant than direct host engraftment as the effects of MSCs continue to appear *in vivo*, even after the cells are displaced or resorbed.

Another mechanism by which they exert their effect is immunomodulation. MSCs inhibit the differentiation of immature monocytes into dendritic cells, which are responsible for antigen presentation to naïve T cells. T cells secrete interferon-α and IL-4, which are responsible for inflammation. MSCs halt the development of T cells in G0/G1 phase and consequently the secretion of interferon-α and IL-4 as well. Finally, they act on natural killer cells making the local environment less susceptible to autoimmune regulation [9]. 

Stem cell therapy in neuropathic pain

The Implementation of Stem Cells in Neuropathic Pain Therapy

Chronic neuropathic pain (NP) is considered to be on the rise, especially with the increase in diabetes prevalence among the United States population [10]. NP is pain that results subsequently from a lesion or disease affecting the somatosensory system. NP was described to have a prevalence of 3% to 17% of the adult life population. Overall, 20% to 25% of all chronic pain is associated with NP. Stem cell therapy is a novel method of treatment that is gaining attention [1]. The classic qualities of NP were noted to be burning, shooting, and sharp [11]. NP can be classified as peripheral or central origin. Trigeminal neuralgia, radiculopathy, polyneuropathy, peripheral nerve injury, and postherpetic neuralgia are all subtypes of peripheral origin NP. On the other hand, subtypes of central origin NP include brain injury, multiple sclerosis, spinal cord injury, and central poststroke pain. All of the previously mentioned subtypes accompany chronic NP that consequently follow the lesion or the disease [1]. The cell-based method of treatment was described by Chakravarthy et al. to be a novel therapeutic approach in the chronic pain associated with neuropathies and degenerative joint disease. Replacement of cells that were previously injured has an impactful role along with delivering trophic factors [12]. MSCs have been shown to be capable of self-renewing and have the potential to differentiate into variable cell types including neurons, adipocytes, osteoblasts, and more [13]. As of now, there is no definite treatment of NP syndrome with concurrent promotion of nervous system repair. Despite that, the novel treatment with the use of stem-cell transplant repairs the nervous system instead of isolated palliation. NP related to disorders including sciatic nerve injury, neuropathies associated with diabetes, and spinal cord injury was demonstrated to be successfully treated with stem cell therapy [10].

Stem Cell Therapy in Diabetic Neuropathy

The term diabetic neuropathy refers to the occurrence of clinical signs and symptoms resulting from a dysfunction in the peripheral nerves in patients with diabetes mellitus. Diabetic neuropathy is considered to be the most common complication of diabetes mellitus, with a rate of 30% to 50% of affected individuals [14]. Investigations on the utilization of stem cells in diabetic neuropathy were done in three studies conducted on animal models described by Vadivelu et al., where administration of stem cells was through the intramuscular route in the hind leg. MSCs were used due to their ability to differentiate into multiple cell types and their cytokine secretion ability. Moreover, a recent study suggests that MSCs can promote neurotrophic factors. The loss of these factors was reported to be partly attributable to diabetic neuropathy, making the use of these stem cells an advantageous point. One of these studies has conducted a trial with the use of marrow mononuclear cells, due to the easy accessibility to this type of cells. These studies have concluded that improvement was noted in two to 15 weeks duration following the injury. Furthermore, these studies have reported this novel approach as a successful treatment method for neuropathic associated pain [10]. The neurotrophic factors secreted by stem cells were reported to accommodate neuronal protection along with providing neuronal regeneration [15]. Although not the routine treatment for diabetic neuropathy, the use of MSC treatment is indicated in patients with diabetic foot ulcers, acute relapses, intractable symptoms, and critical limb ischemic disease. Thereby, this novel treatment targets both vascular and neural components. The benefit of using MSC therapy is known to be attributed to the modulation of the immune response by the short-term effects of the paracrine and juxtacrine roles rather than by lesion site engraftment of the MSCs as a long-term effect. Generating anti-inflammatory MSCs has been shown to alleviate diabetic neuropathy pain. In a mice treatment module, MSC therapy has been shown to reduce proinflammatory cytokine concentrations in the mice’s serum [13]. Prior to and after receiving MSC therapy, the mice were evaluated for painful diabetic neuropathy through two established behavioural assays [16]. Growth factor therapy has shown to be beneficial in diabetic neuropathy due to its ability to promote regeneration of neural tissue and angiogenesis, overall improving nerve function. Bone marrow-derived stem cells were found to produce both angiogenic factors along with neurotrophic growth factors, supplementing selective cells in order to sequent the neuronal regeneration process, and having a more beneficial effect than the growth factor treatment. [14].

Stem Cell Therapy in Trigeminal Neuralgia

Trigeminal NP encompasses variable states of diagnosis, this includes trauma resulting in maxillofacial NP, odontalgia which is atypical, and burning mouth syndrome. Trigeminal NP is considered to be a localized pain. Thereby, its patient population forms an ideal group to investigate the innovative novel therapy. NP is associated with poor response to over-the-counter analgesia and opioids, along with moderate pain relief in 40% of patients in response to anticonvulsant medications and tricyclic antidepressants. MSCs were found to exert anti-inflammatory impacts via cytokine secretion, combatting the ongoing inflammation manifesting as the NP. The previously proposed hypothesis has been studied on animal models with trigeminal NP showing a notable reduction in the inflammatory symptoms and promising outcomes [11]. Sacerdote et al. conducted a study on an animal model with hind paw NP. He reported that utilizing MSCs derived from adipose tissue resulted in the reduction of interleukin-1b pro-inflammatory cytokine and increased levels of IL-10, which is an anti-inflammatory cytokine in injured nerve tissue. A notable decrease in mechanical allodynia and the accompanied thermal hyperalgesia was noticed [17]. MSC therapy administration was previously evaluated and well established to be safe in trials involving both humans and animal models. No abnormal transformations were reported on the cranial nerve-associated nerve physiology. Also, there were no changes in the distributed area of sensation for the trigeminal nerve branches (V2 and V3) and lastly, no notable changes in the injection site. In addition, there were no abnormal reports on motor nerve abnormalities of the face or the jaw involving the seventh cranial nerve or the trigeminal nerve’s motor branch respectively [11].

Stem Cell Therapy in Spinal Cord Injury

Two experimental studies were conducted on mouse models to study spinal cord injury treatment with the use of stem cell therapy. Embryonic stem cell oligosphere culture derivatives such as oligodendrocyte progenitor cells were selected to be utilized by one study group to cause remyelination in the lesioned nerve leading to NP inhibition. Neuregulin was downregulated afterwards through an interfered RNA. Thereafter, the myelinating process was noted to be reduced along with an increment in the allodynia functional measures. The other study used nanoparticles in correlation with co-cultured human stem cells that are derived from adipose tissue. Both *in vivo* and *in vitro* studies resulted in an increase in the expansion and self-renewing process of the administered stem cells. This was especially noted in the GABAergic neurons with a reported significant decrease in the inflammatory cells and the inflammatory mediators along with allodynia improvement after an interval duration of four weeks [10].

Stem cell therapy in discogenic back pain

Chronic low back pain affects 68% of adults older than the age of 60 worldwide. Stem cell therapy has shown beneficial results as an alternative to conventional regimens in the management of degenerative disc disease (DDD). The objective of stem cell therapy is to restore the disc’s cellularity and minimization of the inflammatory response [18].

The causes of disc degeneration are multifactorial; they involve aging, smoking, genetics, nutritional factors, mechanical injury, and comorbidities. Normally, the amount of water and proteoglycan content of the disc increases from the outer annulus fibrosus (AF) to the inner nucleus pulposus. Opposingly, the amount of collagen decreases in the disc from out to in [18,19]. In DDD histology, there is a progressive loss of the transition zone between AF and nucleus pulposus over years. This is due to a change from collagen type II to collagen type I, which is synthesized by the nucleus pulposus. This eventually leads to dehydration and loss of proteoglycans [20]. Disc narrowing, which can be observed radiographically, can be caused by many mechanisms such as matrix metalloprotease-mediated disc degeneration, diminished disc nutrition, etc. [18].

The features of DDD are osteophytes, joint space narrowing, and end plate sclerosis, eventually leading to nerve root compression resulting in symptoms of pain and numbness. Angiogenesis advances from the periphery, eventually extending centrally into the nucleus pulposus, causing discogenic pain [18,21]. Both non-surgical and surgical interventions have failed to manage this condition effectively. A controlled study which was done on 1,450 patients targeting return to work (RTW) as an outcome measure revealed that 67% of the control group had RTW within two years but only 26% of patients could RTW after two years following intervention by fusion surgery [22].

Stem cell therapy restores the cellularity of the IVD and reduces inflammatory mediators. Patient selection plays a pivotal role in the success of stem cell therapy. This intervention can improve the overall outcome in patients who fail to respond to conservative treatment or are in the early stages of DDD [22]. Patients with a disability, as proven by functional scores such as a Pfirrmann grading of Grade III or IV on MRI, and moderate chronic back pain are considered as ideal candidates for the therapy in many studies [23].

Stem cell therapy is currently using stem cells from other sources, autogenic or allogenic in origin or primary cells harvested from the IVD. Nucleus pulposus progenitor cells (NPPC), AF specific progenitor cells, autologous IVD cells, iPSCs, autologous chondrocytes, MSCs (derived from adipose tissue, bone marrow or umbilical cord Wharton's jelly) and embryonic stem cells are some of the different cell lines that have been used as stem cells for discogenic back pain. The different cell lines differ in their characteristics depending on the origin tissue [18].

Harsh environments such as nutrient scarcity, acidic condition and low cellularity make utilization of undifferentiated stem cells a major challenge. The microenvironment of the cultivation culture determines their effectiveness and production of stem cell phenotypes. Stem cell priming with growth factors or different environmental factors such as glucose, oxygen, etc., has been rigorously researched by *in vitro* and *in vivo* studies [18]. Synergistic effect in making a favorable environment has been seen in* in vitro* studies that have combined IVD and MSCs. Inflammatory cytokines and matrix degenerating enzyme-related genes were suppressed in an *in vitro* study that combined human MSCs with rat IVD-NP cells. Similar effects were seen in some* in vivo* studies, with animal models using MSCs. 81% to 91% improvement in MRI signal intensities were noted in post-nucleotomy rabbit models due to suppression of type 1 collagen formation with the use of MSCs injection compared to sham-treated discs which showed 67% to 60%. Reduced disc tissue degeneration, microenvironment catabolism, recovered disc height and decreased pain has been evident in the growing number of studies. Culturing of human MSCs *in vitro* has proven that many of these effects are reproducible. Improvement in pain and function has been noted in limited clinical studies that have been done. Both animal and human studies have successfully shown evidence supporting disc regeneration and at least partial recovery in addition to safety and feasibility. To collect more data on human benefits, further clinical studies are required [24].

Gene therapy has been advanced in order to overcome the shortcomings of conventional methods. Over direct delivery of proteins, gene therapy has advantages such as enhanced efficacy and sustained anti-inflammatory factors and growth factors synthesis endogenously [18]. Image-assisted percutaneous injection through the AF has been used conventionally, although it has raised some safety concerns. Alternative routes of administration are through the pedicles (transpedicular approach) and use of delivery vehicles [25]. Delivery vehicles, hydrogels have been utilized to overcome retention issues and provide additional support for cell survival and phenotype retention. Scaffolding materials, such as hyaluronan, fibrin, and atelocollagen have been developed to improve efficiency of stem cell delivery into degenerated IVDs [18].

Stem cell therapy in osteoarthritis

Osteoarthritis (OA) remains to constitute a large burden to healthcare and negatively impact the quality of life; along with other conditions ranked as 11th highest contributors of global disability [26]. The estimated prevalence of problematic hip/knee OA is approximately 242 million globally; 3.8% when accounting for decreased quality of life (QoL) and societal burden [27,28]. Knee OA (KOA) carries a higher incidence when compared to other joints (i.e., hip), increasing to 60% among the obese population [29]. Of note, a population-based cohort study published in 2008 estimated a 45% lifetime risk of symptomatic knee-osteoarthritis [30]. Specifically, KOA was shown to be affecting nine million adults (over the age of 45 years) in the United States with symptoms ranging from moderate to severe [31]. With the escalation of an aging population, sedentary lifestyle, and obesity, the number of patients affected with KOA is undoubtedly on the rise.

The concept of age-related joint degeneration in the event of KOA has been substituted by other theories and factors. In fact, KOA has been shown to be the end result of a chronic interplay between heterogeneous systemic and local reactions. Systemic factors include age, race, ethnicity, and diet, while local ones include body-mass-index (BMI), trauma history, occupational, and mechanical factors [32]. The aforementioned factors, along with indigenous biologic factors (i.e., cytokine homeostasis) result in trivial changes at the knee joint, particularly at the area interspacing between the subchondral bone and the articular cartilage, which becomes evident over time [33]. Owing to this effect, an increase in bone mass and trabecular thickness ensues with reduced ability to withstand stress and compression impact on cartilage; the stage at which chondrocyte cell senescence occurs [30]. Exhibited degeneration of the articular cartilage is a pathognomic feature for OA associated with bone remodeling, osteophyte formation, capsular distribution, and periarticular muscle atrophy [30]. The complexity of cartilage is derived from multiple building blocks and constituents such as chondrocytes type II, collagen, proteoglycan, and an abundantly hydrophilic extracellular matrix containing highly complexed meshwork of cytokines and growth factors secreted by surrounding synovial cells and chondrocytes [30]. Prolonged exposure to stressors including reactive oxygen species (ROS) and nitric oxide (NO) in turn trigger macrophages and nuclear factor kappa-light-chain-enhancer of activated B cells (NF-kβ). They play an important role in immune regulation and activation of cytokines which are associated with inflammation and ultimately cause disruption of homeostasis in the synovial fluid [30]. Other important associated inflammatory molecules include IL-1 β, TNF-α, INF- γ, TGFβ, MMP-9, and MMP-13, all of which contribute to pathologic hallmarks in the pathogenesis of OA [34].

Current conventional therapy of OA is mainly focused on providing symptomatic control over hindering disease course progression. Commonly used radiographic grading systems for KOA include Kellgren-Lawrence (K/L). Along with exercise therapy, physical therapy, and weight reduction to strengthen adjacent muscles, current pharmacologic treatment includes acetaminophen, non-steroidal anti-inflammatory drugs (NSAIDs), gabapentin, pregabalin, and opioids, which are used for K/L grade 0-1. Other means include valgus directing force bracing [32,35]. Intra-articular (IA) corticosteroid injections are commonly prescribed as a symptomatic treatment for grade ≥ 2 upon which osteophyte formation, joint space narrowing, subchondral sclerosis, and deformity of the joint are evident [32,35]. Subsequently, total knee arthroplasty (TKA) becomes an option for K/L grade 3-4. It is an invasive procedure associated with a significant number of complications. It was shown that 20% of patients who underwent TKA will have persistent knee pain with a possible need for TKA revision and is subject to further risk and morbidity including loss of function within a year after the procedure [34]. Moreover, with the uncertainty of progression and symptomatic treatment following TKA, it was estimated that 61% of medical expenses are spent on TKA procedures [32]. 

Compared to conventional therapy which provides symptomatic treatment, multiple randomized control trials (RCTs) have demonstrated promising potential for stem cell therapies tackling OA morbidity and healthcare burden. The concept of stem cells arises from multipotent cells which have the ability to differentiate into different cell types and possess auto-regeneration according to the body’s needs. These cells can be readily extracted from tissues in the body including the bone marrow, adipose tissue, and the synovium [36]. Of note, MSCs are adult stem cells (ASCs) derived from the mesodermal origin and have the ability to differentiate into different connective tissue cells including osteocytes, adipocytes, and chondrocytes [37]. *In vitro* MSCs were shown to have the ability to differentiate into chondrocytes and enhance the proliferation of resident progenitor cells *in vivo*. With the help of growth factors and extracellular matrix proteins, MSCs demonstrated the ability to create a repair microenvironment that could explain the postulated theory of associated pain reduction and the termination of the disease progression cycle [38]. Subsequently, this method of autologous repairing gives rise to the activation of senescent, metabolically active chondrocytes’ ability to repair damaged tissue [38]. Trials suggested that transplanted MSCs in the joint are activated and subsequently expressing anabolic genes such as the Indian hedgehog and other ‘hit and run’ genes that enhance collagen type synthesis, analgesic peptide transcription, and the production of various anti-inflammatory cytokines and analgesic proteins [39,40]. The aforementioned disease-modifying properties build an evidence-based tool for MSC therapy in the context of OA disease progression and stabilization [36,40].

Performed clinical trials have subjectively depended on different scoring systems in assessing the effect of engrafted MSCs including the Knee Injury and Osteoarthritis Outcome Score (KOOS), Western Ontario, and McMaster Universities Arthritis Index (WOMAC), and the Visual Analog Scale (VAS). According to Buzaboon et al., MSCs treatments demonstrated pain reduction and improved knee joint function post-intervention from baseline according to different parameters such as KOOS, VAS, and WOMAC. Most trials have demonstrated a peak in positive effects occurring between the sixth and 12th months following treatment [33,39,41]. However, subjectively assessed results were not statistically significant in most trials selected in the study. This was assumed to be due to the late intervention with MSCs to treat OA, which was probably at an advanced stage. The theory is that MSC implantation is more effective in OA when provided at early stages of disease [33]. Nonetheless, MSCs implantation in OA has been shown to be a more superior approach to conventional therapy. This can be explained by the fact that MSCs treatment can hinder inflammation, restore vital tissue components and damaged cartilage, and the negligible number of serious local or generalized systemic adverse events and complications. However, with the early promising results of MSCs therapy in OA, many questions regarding precise mechanisms remain unexplored. There is still a need for further clinical trials and studies to be performed at the early stages of OA (K/L Grade 0-2), examining different cell culture preparation, and exploring different dosing intervals, frequency of therapy, and appropriate delivery method.

Stem cell therapy in musculoskeletal disease

The World Health Organization (WHO) has enlisted musculoskeletal disease as one of the most common causes of severe long-term pain and physical disability. In 2016, it comprised the second-highest global volume of years lived with disability [42]. Musculoskeletal conditions may involve bones, muscle, tendons, cartilage, ligaments, joints, with pain often being the first presenting complaint. With increasing life expectancy, the burden of musculoskeletal diseases is on the rise. However, the treatment modalities are limited to managing the symptoms rather than curing it. The rapid development of regenerative medicine and stem cell therapy offers hope.

MSCs can rapidly divide and repair damaged cartilage, making them suitable to treat conditions such as tendon, ligament, and cartilage damage. Stem cells are easy to extract, prepare, and inject making them suited for outpatient settings. MSCs are prepared via centrifugation of harvested tissue, and then separated to isolate MSC dense fluid. This fluid is then injected into the autologous therapeutic tissue of interest such as joint, tendon, cartilage, or intervertebral disc. Bone marrow aspirate concentrate (BMAC) has been commonly used for the treatment of musculoskeletal conditions, but there are reports of using stem cells derived from peripheral blood stem cells (PBSCs) [43].

There are limited data evaluating the safety and efficacy of stem cells in humans for musculoskeletal disease. In a systematic review by Law et al., bone marrow-derived MSCs were found to be safe and feasible for chronic patellar tendinopathy and femoral head necrosis, but three out of eight studies did not reach statistical significance [44]. In a five-year study conducted by Pascual-Garrido et al., stem cell therapy showed improvement in clinical scores, pain, as well as improvement in ultrasound grade in seven out of eight patients with chronic patellar tendinopathy. Maximal improvement was seen at two years and was maintained at five years [45]. In patients with femoral head necrosis, core decompression plus BMAC resulted in better pain relief than decompression alone as assessed by Harris Hip Score, but it did not reach statistical significance. However, the injection of PBSCs improved pain function, imaging and other patient-reported outcomes over control in patients with osteonecrosis of femoral head [46]. Most of the studies conducted involved a small sample size. Despite this limited evidence supporting the use of stem cells in musculoskeletal conditions, their use continues to grow in the United States and other parts of the world. Furthermore, there has been a recent interest in anterior cruciate ligament regeneration using MSCs. Although initial success has been achieved in animal models, its potential is yet to be realized in humans. There is a need for long-term randomized controlled trials before stem cell therapy gains wide acceptance in treatment of musculoskeletal diseases.

Side effects/risks of stem cell therapy

Stem cell transplantation is considered a safe option for the above-mentioned medical conditions. Many researchers have reported no adverse outcomes among patients following stem cell therapy. Lalu et al. conducted a meta-analysis on 1012 patients who underwent stem cell transplantation for multiple medical conditions such as inflammatory bowel disease, stroke, cardiomyopathies, cardiac ischemia and reported no adverse events during and 90 months after the procedure [47]. Other studies have reported mild adverse effects after the procedure among which include nausea, vomiting, infections, and endocrine dysfunction [48]. Yobu et al. conducted a meta-analysis on the use of MSC transplantation for the management of knee osteoarthritis. This study comprised 582 patients and reported that the majority of adverse events were minor such as pain at the site of injection, swelling of knee joint, reduced range of motion, infections. Some patients manifested small or large bowel obstruction which was managed accordingly [49]. El-Badaway et al. conducted a meta-analysis on the efficacy of stem cell therapy used for the management of diabetic patients. Their study results reported minor adverse effects such as nausea and abdominal pain in 21.7% of patients [50].

However, some clinicians have reported some serious adverse events associated with it. An important adverse effect of stem cell therapy is the risk of tumor formation secondary to the malignant transformation of MSCs. Certain factors which account for tumor formation secondary to stem cell therapy consist of expression of oncogenic tumor cell markers, chromosomal instability or increased telomerase activity. Other factors which have been observed to play a role in tumor progression include the inhibitory effect of MSC on immune regulatory cells of the body. Some researchers have proven that if there are any existing tumor cells in the body, they can escape immune surveillance after the commencement of stem cell therapy [51]. If the MSC exhibits disease memory or has been excised from diseased tissue it can exhibit the expression of inflammatory markers which can lead to disease expression in transplanted patients. There have been previous studies that have demonstrated the development of malignancy after stem cell transplantation [52]. Pan et al. conducted a study by transplanting transformed MSC derived from human bone marrow and liver in immunodeficient mice. They observed the development of sarcoma-like tumors and concluded that efficient screening of MSC cultures can prevent tumorigenesis as an adverse event [53].

## Conclusions

The potential of human-based PSC therapy in a wide array of applications is promising. Clinical application of stem cell-based therapy has been described in approximately 14 diseases and trauma-related implications and is currently investigated by multiple clinical trials. The utilization of stem cell therapy directed towards the regeneration of certain cell types including senescent cells and other components of organ tissues is challenging yet very promising. Ongoing clinical trials have shown positive outcomes of stem cell therapy in treating chronic diseases associated with significant morbidity and reduced QoL. In this review article, we shed light on the current advancement of stem cell therapy in pain management. Along with trophic factors and other anabolic effects of stem cell therapy, the pain of chronic diseases including DM-associated neuropathic pain, OA, back pain, ligamentous pain, and other injuries can be effectively managed with this modality reflecting into decreased healthcare burden and improvement of QoL. In addition, their effect can be reasonably fast, and the long duration of their effect adds to their advantages. The strengths of this review include the fact that there are many supporting studies and reviews which encourage the advancement of stem cell therapy, the only limitation is that majority of these studies are in their earlier stages. Despite the fact that utilization of stem cell therapy is still in an early stage and most major studies are still at their preclinical phase, the prospect of this approach is very assuring given the advancement in technology and the increasing number of clinical studies and evidence. Future studies should focus on human-based clinical trials since the results of animal trials may not entirely be applicable in humans. 
